# Supplementary material for: Bilateral JNK activation is a hallmark of interface surveillance and promotes elimination of aberrant cells
Source: eLife. 2023 Feb 6;12:e80809. doi: 10.7554/eLife.80809 (PMC9917460; doi:10.7554/eLife.80809)
Supplement: Supplementary file 1. — Table listing fly strains used in this study. [file elife-80809-supp1.docx]

**Table S1 Fly strains**

| **Genotype** | **Chrom.** | **Source** |
| --- | --- | --- |
| *w^118^* | I | David Bilder |
| *hsflp^122^* | I | Iswar Hariharan |
| *act>y^+^>GAL4, UAS-GFP/TM6b* | III | David Bilder |
| *UAS-fkh-3xHA* | I | Martin Juenger |
| *hsflp^122^; Dad^4^-LacZ/CyO* | I,II,III | Giorgios Pyrowolakis |
| *UAS-tkv^CA^* | I | BDSC 36537 |
| *tub-miniCic-mCherry (or miniCic-mCherry)* | II | Romain Levayer |
| *tub-miniCic-mScarlett (or miniCic-mScarlett)* | II | Romain Levayer |
| *UAS-Egfr^CA^/TM6c* | III | BDSC 59843 |
| *UAS-p35* | II | David Bilder |
| *UAS-p35* | III | David Bilder |
| *UAS-ey* | II | BDSC 6294 |
| *UAS-arm^S10^* | I | Suzanne Eaton |
| *UAS-ci-HA* | III | BDSC 32570 |
| *UAS-tkv-RNAi (TRiP.HMS02185)* | II | BDSC 40937 |
| *TRE-RFP* | II | Dirk Bohmann |
| *puc^A251.1F3^ >LacZ/TM6c* | III | BDSC 11173 |
| *UAS-bsk^DN^* | I | BDSC 6409 |
| *UAS-myc-HA* | III | BDSC 64759 |
| *FRT82B ubi-GFP, RpS3^Plac92^/TM6c* | III | BDSC 5627 |
| *UAS-wts RNAi (101055/KK)* | II | VDRC 111002 |
| *en-GAL4, UAS-GFP* | II | David Bilder |
| *brk-GAL4; UAS-CD8-GFP, LexO-mCherry-CAAX/SM5-TM6b; LexΟ-tkv^CA^ /SM5-TM6b* | I,II,III | Giorgios Pyrowolakis |
| *tub-GAL80^ts^* | III | BDSC 7018 |
| *UAS-Ras^V12^* | II | Helena Richardson |
| *UAS-Ras^V12^* | III | BDSC 4847 |
