# Supplementary material for: Bilateral JNK activation is a hallmark of interface surveillance and promotes elimination of aberrant cells
Source: eLife. 2023 Feb 6;12:e80809. doi: 10.7554/eLife.80809 (PMC9917460; doi:10.7554/eLife.80809)
Supplement: Supplementary file 2. — Table listing detailed genotypes per figure panels [file elife-80809-supp2.docx]

**Table S2 Detailed genotypes**

| **Figure** | **Genotype** | **Heatshock** | **Analysis after:** |
| --- | --- | --- | --- |
| 1 C,J,N | *hsflp^122^/+; +/+;*  *act>y^+^>GAL4, UAS-GFP/+* | 9.5 min,  8.5 min | 30 h 25 °C |
| 1 E,K,O | *UAS-fkh-3xHA/hsflp^122^; +/+;*  *act>y^+^>GAL4, UAS-GFP/+* | 9.5 min,  8.5 min | 30 h 25 °C |
| 1 G,L | *hsflp^122^/+; +/+;*  *act>y^+^>GAL4, UAS-GFP/UAS-tkv^CA^* | 9.5 min | 30 h 25 °C |
| 1 I,M | *hsflp^122^/+; UAS-tkv-RNAi/+;*  *act>y^+^>GAL4, UAS-GFP/+* | 9.5 min | 30 h 25 °C |
| 1 P | *UAS-fkh-3xHA/hsflp^122^; UAS-p35/+; act>y^+^>GAL4, UAS-GFP/+* | 8.5 min | 30 h 25 °C |
| S1.2 A,C,F,G | *hsflp^122^/+; +/+;*  *act>y^+^>GAL4, UAS-GFP/+* | 9.5 min, | 30 h 25 °C |
| S1.2 B | *UAS-fkh-3xHA/hsflp^122^; +/+;*  *act>y^+^>GAL4, UAS-GFP/+* | 9.5 min | 30 h 25 °C |
| S1.2 D,K | *hsflp^122^/+; UAS-ey/+;*  *act>y^+^> GAL4, UAS-GFP/+* | 9.5 min,  8.5 min | 30 h 25 °C |
| S1.2 E | *hsflp^122^/+; TRE-RFP/Dad^4^-LacZ;*  *act>y^+^>GAL4, UAS-GFP/+* | 9.5 min | 30 h 25 °C |
| S1.2 H | *hsflp^122^/+; tub-miniCic-mCherry/+;*  *act>y^+^>GAL4, UAS-GFP/+* | 9.5 min | 30 h 25 °C |
| S1.2 M | *UAS-arm^S10^/ hsflp^122^; +/ +;*  *act>y^+^>GAL4, UAS-GFP/+* | 9.5 min | 30 h 25 °C |
| S1.2 O | *hsflp^122^/+; +/+;*  *act>y^+^>GAL4, UAS-GFP/UAS-ci-HA* | 9.5 min | 54 h 18 °C |
| S1.2 Q | *hsflp^122^/+; tub-miniCic-mCherry/+;*  *act>y^+^>GAL4, UAS-GFP/UAS-Egfr^CA^* | 9.5 min | 30 h 25 °C |
| S1.3 B | *hsflp^122^/+; +/+;*  *act>y^+^>GAL4, UAS-GFP/UAS-p35* | 8.5 min | 30 h 25 °C |
| S1.3 C | *hsflp^122^/+; UAS-ey/+;*  *act>y^+^> GAL4, UAS-GFP/+* | 8.5 min | 30 h 25 °C |
| S1.3 D | *hsflp^122^/+; UAS-ey/+;*  *act>y^+^>GAL4, UAS-GFP/UAS-p35* | 8.5 min | 30 h 25 °C |
| 2 A,D | *hsflp^122^/+; TRE-RFP/+;*  *act>y^+^>GAL4, UAS-GFP/ +* | 8.5 min,  25 min | 30 h 25 °C |
| 2 C,F | *UAS-fkh-3xHA/hsflp^122^; TRE-RFP/+;*  *act >y^+^>GAL4, UAS-GFP/+* | 8.5 min,  25 min | 30 h 25 °C |
| 2 G | *hsflp^122^/+; TRE-RFP/Dad^4^-LacZ;*  *act>y^+^>GAL4, UAS-GFP/+* | 9.5 min | 30 h 25 °C |
| 2 H | *hsflp^122^/+; TRE-RFP/Dad^4^-LacZ;*  *act>y^+^>GAL4, UAS-GFP/ UAS-tkv^CA^* | 9.5 min | 30 h 25 °C |
| S 2.1 A,C | *hsflp^122^/+; TRE-RFP/UAS-ey;*  *act>y^+^>GAL4, UAS-GFP/+* | 8.5 min,  25 min | 30 h 25 °C |
| S2.1 E,G,I | *hsflp^122^/+; TRE-RFP/+;*  *act>y^+^>GAL4, UAS-GFP/+* | 9.5 min | 30 h 25 °C |
| S2.1 F | *hsflp^122^/+; TRE-RFP/+;*  *act>y^+^>GAL4, UAS-GFP/UAS-Egfr^CA^* | 9.5 min | 30 h 25 °C |
| S2.1 H | *UAS-arm^S10^/hsflp^122^; TRE-RFP/+;*  *act>y^+^>GAL4, UAS-GFP/+* | 9.5 min | 30 h 25 °C |
| S2.1 J | *hsflp^122^/+; TRE-RFP/+;*  *act>y^+^>GAL4, UAS-GFP/UAS-ci-HA* | 9.5 min | 54 h 18 °C |
| S2.2 A | *hsflp^122^/+; +/+; puc^A251.1F3^-LacZ /act>y^+^>GAL4, UAS-GFP/+* | 10 min | 30h 25 °C |
| S2.2 B | *UAS-fkh-3xHA/hsflp^122^; +/+ ; puc^A251.1F3^>LacZ/act>y^+^>GAL4, UAS-GFP* | 10 min | 30 h 25 °C |
| S2.2 C | *hsflp^122^/+; UAS-ey /+; puc^A251.1F3^>LacZ/act>y^+^>GAL4, UAS-GFP/ +* | 10 min | 30 h 25 °C |
| S2.2 D | *hsflp^122^/+; + /+; puc^A251.1F3^-LacZ/act>y^+^>GAL4, UAS-GFP/UAS-Ras^V12^* | 10 min | 30 h 25 °C |
| 3 A,D | *UAS-bsk^DN^/hsflp^122^; TRE-RFP/+;*  *act>y^+^>GAL4, UAS-GFP/+* | 9.5 min  25 min | 30 h 25 °C |
| 3 B,E | *hsflp^122^/+; TRE-RFP/UAS-ey;*  *act>y^+^>GAL4, UAS-GFP/+* | 9.5 min  25 min | 30 h 25 °C |
| 3 C,F | *UAS-bsk^DN^/hsflp^122^; TRE-RFP/UAS-ey; act>y^+^>GAL4, UAS-GFP/+* | 9.5 min  25 min | 30 h 25 °C |
| S3 A,B | *hsflp^122^/+; TRE-RFP/+;*  *act>y^+^>GAL4, UAS-GFP/+* | 9.5 min  25 min | 30 h 25 °C |
| S3 C,E | *hsflp^122^/+; TRE-RFP/+;*  *act>y^+^>GAL4, UAS-GFP/UAS-tkv^CA^* | 9.5 min  25 min | 30 h 25 °C |
| S3 D,F | *UAS-bsk^DN^/hsflp^122^; TRE-RFP/+;*  *act>y^+^>GAL4, UAS-GFP/UAS-tkv^CA^* | 9.5 min  25 min | 30 h 25 °C |
| 4 A | *hsflp^122^/+; TRE-RFP/+;*  *act>y^+^>GAL4, UAS-GFP/+* | 9.5 min | 30 h 25 °C |
| 4 B | *hsflp^122^/+; TRE-RFP/+;*  *act>y^+^>GAL4, UAS-GFP/UAS-myc-HA* | 9.5 min | 30 h 25 °C |
| 4 D | *hsflp^122^/+; TRE-RFP/+; FRT82B ubi-GFP, RpS3^Plac92^/FRT82B* | 1 h | 30 h 25 °C |
| S4.1 A | *hsflp^122^/+; TRE-RFP/+;*  *act>y^+^>GAL4, UAS-GFP/+* | 9.5 min | 54 h 25 °C |
| S4.1 B | *hsflp^122^/+; TRE-RFP/+;*  *act>y^+^>GAL4, UAS-GFP/UAS-wts RNAi* | 9.5 min | 54 h 25 °C |
| S4.2 A | *hsflp^122^/+; TRE-RFP/UAS-ey;*  *act>y^+^>GAL4, UAS-GFP/+* | 9.5 min | 30 h 25 °C |
| S4.2 B | *UAS-bsk^DN^/hsflp^122^; TRE-RFP/UAS-ey; act>y^+^>GAL4, UAS-GFP/+* | 9.5 min | 30 h 25 °C |
| S4.2 C | *hsflp^122^/+; TRE-RFP/+;*  *act>y^+^>GAL4, UAS-GFP/UAS-tkv^CA^* | 9.5 min | 30 h 25 °C |
| S4.2 D | *UAS-bsk^DN^/hsflp^122^; TRE-RFP/+;*  *act>y^+^>GAL4, UAS-GFP/UAS-tkv^CA^* | 9.5 min | 30 h 25 °C |
| S4.2 E | *hsflp^122^/UAS-bsk^DN^; en-GAL4/+;*  *act>y^+^>UAS-GFP, LexO-mCherry /TM6c* | 9.5 min | 30 h 25 °C |
| S4.2 F | *hsflp^122^/UAS-bsk^DN^; en-GAL4/+;*  *act>y^+^>UAS-GFP, LexO-mCherry; LexO-tkv^CA^/act>>VH2* | 9.5 min | 30 h 25 °C |
| 5 A | *UAS-fkh-3xHA/hsflp^122^; TRE-RFP/+;*  *act >y^+^>GAL4, UAS-GFP/+* | 8.5 min | 30 h 25 °C |
| 5 E | *hsflp^122^/+; TRE-RFP/+;*  *act>y^+^>GAL4, UAS-GFP/UAS-tkv^CA^* | 9.5 min | 30 h 25 °C |
| 5 G | *hsflp^122^/UAS-bsk^DN^; TRE-RFP/+;*  *act> y^+^>GAL4, UAS-GFP/UAS-tkv^CA^* | 9.5 min | 30 h 25 °C |
| 5 I | *hsflp^122^/UAS-bsk^DN^; en-GAL4/+;*  *act> y^+^>UAS-GFP, LexO-mCherry /TM6c* | 9.5 min | 30 h 25 °C |
| 5 J | *hsflp^122^/UAS-bsk^DN^; en-GAL4/+;*  *act> y^+^>UAS-GFP, LexO-mCherry; LexO-tkv^CA^/act>>VH2* | 9.5 min | 30 h 25 °C |
| S5 E | *hsflp^122^/UAS-fkh-3xHA; tub-GAL80^ts-20^/Sp or +; act>y^+^>GAL4, UAS-GFP/Ly* | 11 min | 30 h 18° C  18 h 30 °C |
| S5 F | *hsflp^122^/UAS-fkh-3xHA; tub-GAL80^ts-20^/Sp or +; act>y^+^>GAL4, UAS-GFP/Ly* | 11 min | 30 h 30 °C |
| S6.1 C | *UAS-fkh-3xHA/hsflp^122^; tub-miniCic-mCherry/+; act>y^+^>GAL4, UAS-GFP/+* | 10 min | 30 h 25 °C |
| S6.1 D | *UAS-fkh-3xHA/hsflp^122^; tub-miniCic-mCherry/+;*  *act>y^+^>GAL4, UAS-GFP/UAS-Ras^V12^* | 10 min | 30 h 25 °C |
| S6.1 G | *hsflp^122^/+; tub-miniCic-mCherry/UAS-ey; act>y^+^>GAL4, UAS-GFP/ +* | 10 min | 30 h 25 °C |
| S6.1 H | *hsflp^122^/+; tub-miniCic-mCherry/UAS-ey; act>y^+^>GAL4, UAS-GFP/UAS-Ras^V12^* | 10 min | 30 h 25 °C |
| S6.2 A | *hsflp^122^/+; tub-miniCic-mCherry/+; act>y^+^>GAL4, UAS-GFP/UAS-Ras^V12^* | 10 min | 30 h 25 °C |
| S6.2 B | *hsflp^122^/+; tub-miniCic-mCherry/UAS-ey; act>y^+^>GAL4, UAS-GFP/ +* | 10 min | 30 h 25 °C |
| S6.2 C | *hsflp^122^/+; tub-miniCic-mCherry/UAS-ey; act>y^+^>GAL4, UAS-GFP/UAS-Ras^V12^* | 10 min | 30 h 25 °C |
| 7 A,D | *hsflp^122^/+; +/+;*  *act>y^+^>GAL4, UAS-GFP/UAS-Ras^V12^* | 9.5 min  10 min | 30 h 25 °C |
| 7 B | *hsflp^122^/+; TRE-RFP/+;*  *act>y^+^>GAL4, UAS-GFP/UAS-Ras^V12^* | 9.5 min | 30 h 25 °C |
| 7 C | *hsflp^122^/+; +/+;*  *act>y^+^>GAL4, UAS-GFP/+* | 10 min | 30 h 25 °C |
| 7 E | *UAS-fkh-3xHA/hsflp^122^; +/+;*  *act>y^+^>GAL4, UAS-GFP/+* | 10 min | 30 h 25 °C |
| 7 F | *UAS-fkh-3xHA/hsflp^122^; +/+;*  *act>y^+^>GAL4, UAS-GFP/UAS-Ras^V12^* | 10 min | 30 h 25 °C |
| S7.1 B,F | *hsflp^122^/+; tub-miniCic-mCherry/+; act>y^+^>GAL4, UAS-GFP/UAS-Ras^V12^* | 10 min | 30 h 25 °C |
|  |  |  |  |
